# Tau-PET imaging and blood biomarkers reveal early tauopathy in special operations forces exposed to repetitive blast

**DOI:** 10.1093/braincomms/fcag070

**Published:** 2026-03-13

**Authors:** Shamantha J Lora, Shawn G Rhind, Sarah E Watling, Lucas Narciso, Jerry Warsh, Oshin Vartanian, Tina McCluskey, Rachel F Tyndale, Maria Carmela Tartaglia, Maria Y Shiu, Isabelle Vallée, Mike Crouzat, Iain Vergie, Neil Vasdev, Isabelle Boileau

**Affiliations:** Brain Health Imaging Centre, Centre for Addiction and Mental Health, Toronto, ON M5T 1R8, Canada; Institute of Medical Sciences, University of Toronto, Toronto, ON M5S 3H2, Canada; Defence Research and Development Canada, Toronto Research Centre, Toronto, ON M3K 2C9, Canada; Faculty of Kinesiology and Physical Education, University of Toronto, Toronto, ON M5S 2W6, Canada; Brain Health Imaging Centre, Centre for Addiction and Mental Health, Toronto, ON M5T 1R8, Canada; Institute of Medical Sciences, University of Toronto, Toronto, ON M5S 3H2, Canada; Brain Health Imaging Centre, Centre for Addiction and Mental Health, Toronto, ON M5T 1R8, Canada; Institute of Medical Sciences, University of Toronto, Toronto, ON M5S 3H2, Canada; Campbell Mental Health Research Institute, Centre for Addiction and Mental Health, Toronto, ON M5T 1R8, Canada; Department of Psychiatry, University of Toronto, Toronto, ON M5T 1R8, Canada; Brain Health Imaging Centre, Centre for Addiction and Mental Health, Toronto, ON M5T 1R8, Canada; Institute of Medical Sciences, University of Toronto, Toronto, ON M5S 3H2, Canada; Campbell Mental Health Research Institute, Centre for Addiction and Mental Health, Toronto, ON M5T 1R8, Canada; Department of Psychiatry, University of Toronto, Toronto, ON M5T 1R8, Canada; Pharmacology and Toxicology, University of Toronto, Toronto, ON M5S 3K3, Canada; Defence Research and Development Canada, Toronto Research Centre, Toronto, ON M3K 2C9, Canada; Department of Psychology, University of Toronto, Toronto, ON M5S 2E5, Canada; Brain Health Imaging Centre, Centre for Addiction and Mental Health, Toronto, ON M5T 1R8, Canada; Campbell Mental Health Research Institute, Centre for Addiction and Mental Health, Toronto, ON M5T 1R8, Canada; Institute of Medical Sciences, University of Toronto, Toronto, ON M5S 3H2, Canada; Campbell Mental Health Research Institute, Centre for Addiction and Mental Health, Toronto, ON M5T 1R8, Canada; Department of Psychiatry, University of Toronto, Toronto, ON M5T 1R8, Canada; Pharmacology and Toxicology, University of Toronto, Toronto, ON M5S 3K3, Canada; Institute of Medical Sciences, University of Toronto, Toronto, ON M5S 3H2, Canada; Tanz Centre for Research in Neurodegenerative Diseases, University of Toronto, Toronto, ON M5S 1A8, Canada; Defence Research and Development Canada, Toronto Research Centre, Toronto, ON M3K 2C9, Canada; National Defence and the Canadian Armed Forces, Canadian Special Operations Forces Command, Ottawa, ON K1N 8T7, Canada; National Defence and the Canadian Armed Forces, Canadian Special Operations Forces Command, Ottawa, ON K1N 8T7, Canada; National Defence and the Canadian Armed Forces, Canadian Special Operations Forces Command, Ottawa, ON K1N 8T7, Canada; Brain Health Imaging Centre, Centre for Addiction and Mental Health, Toronto, ON M5T 1R8, Canada; Institute of Medical Sciences, University of Toronto, Toronto, ON M5S 3H2, Canada; Campbell Mental Health Research Institute, Centre for Addiction and Mental Health, Toronto, ON M5T 1R8, Canada; Department of Psychiatry, University of Toronto, Toronto, ON M5T 1R8, Canada; Azrieli Centre for Neuro-Radiochemistry, Centre for Addiction and Mental Health, Toronto, ON M5T 1R8, Canada; Brain Health Imaging Centre, Centre for Addiction and Mental Health, Toronto, ON M5T 1R8, Canada; Institute of Medical Sciences, University of Toronto, Toronto, ON M5S 3H2, Canada; Campbell Mental Health Research Institute, Centre for Addiction and Mental Health, Toronto, ON M5T 1R8, Canada; Department of Psychiatry, University of Toronto, Toronto, ON M5T 1R8, Canada; Pharmacology and Toxicology, University of Toronto, Toronto, ON M5S 3K3, Canada

**Keywords:** positron emission tomography, [^18^F]Flortaucipir, blast-wave, brain injury, military personnel

## Abstract

Repeated low-intensity blast overpressure exposures, frequently sustained by Special Operations Forces during breaching, combat training, and weapons use, are thought to initiate tau-related neurodegenerative changes that may remain clinically silent for years. The long-term impact of cumulative blast overpressure on brain health is poorly understood, and sensitive biomarkers are needed for early detection and monitoring of subclinical injury in high-risk populations. In this cross-sectional study, 25 actively serving male Canadian Special Operations Forces personnel (mean [SD] age, 43.6 [6.1] years) with ≥16 years of breaching and explosives experience were compared with 10 age-matched Canadian Armed Forces controls (mean [SD] age, 39.8 [6.8] years) with minimal blast exposure. All participants underwent [^18^F]flortaucipir PET imaging to quantify cortical tau deposition, MRI, ultrasensitive digital immunoassay for plasma biomarkers, and comprehensive clinical and neurocognitive testing. Group differences in regional [^18^F]flortaucipir standardized uptake value ratios were assessed using analysis of covariance, and voxelwise *Z*-score mapping identified clusters of elevated tracer uptake (>2 SD above control mean). Linear regression analyses were conducted to examine associations between PET tau signal, plasma biomarkers, cumulative blast exposure and clinical outcomes. Special Operations Forces personnel exhibited significantly higher [^18^F]flortaucipir uptake in the frontal (*P* & 0.022) and temporal cortices (*P* & 0.037) compared with controls. Voxelwise mapping revealed tau clusters in 88% of exposed individuals, with nearly half localized to the frontal cortex. Elevated PET signal correlated with cumulative years of breaching, post-concussive symptoms, sleep disturbance and functional impairment. Plasma biomarkers showed converging evidence of neurodegeneration: brain-derived tau, glial fibrillary acidic protein and amyloid-β42 levels were significantly associated with regional tau PET uptake. A reduced amyloid-β42/40 ratio and elevated phosphorylated tau isoforms further supported early molecular changes consistent with neurodegeneration. Cumulative occupational blast overpressure exposure in Special Operations Forces is associated with frontal-predominant tau deposition and plasma biomarker signatures of astroglial activation, axonal injury and CNS-specific tau release. These convergent imaging and molecular findings support a link between repetitive blast exposure and early-stage tauopathy, and highlight the value of combined tau PET imaging and fluid biomarkers as non-invasive tools for early detection, monitoring, and targeted risk mitigation in blast-exposed populations.

## Teaser

Lora *et al*. reported that repeated low-intensity blast exposure in Special Operations Forces was linked to increased frontal-predominant tau deposition using PET and elevations in plasma biomarkers of astroglial and neuronal injury. These findings highlight the need for enhanced monitoring, personalized risk evaluation and proactive protection of military brain health.

## Graphical Abstract

**Graphical Abstract:**
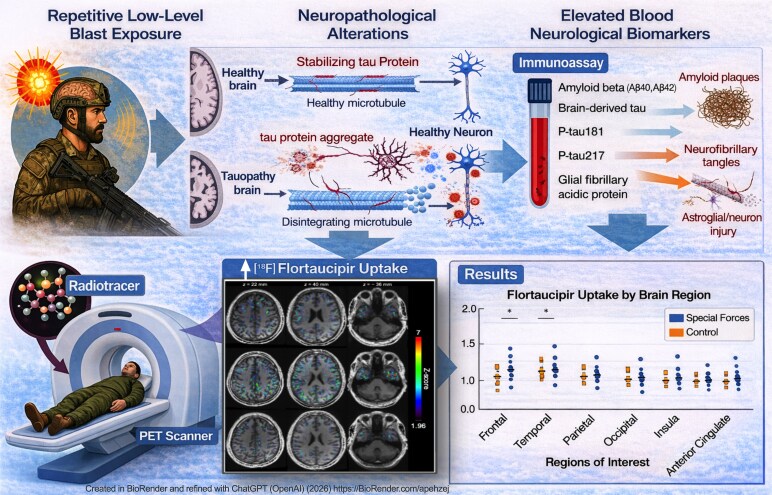

## Introduction

Military personnel, particularly those serving in Special Operations Forces (SOF), are routinely exposed to occupational hazards that threaten brain health and operational readiness.^1,2^ Among the most pervasive is blast overpressure (BOP), the high-energy pressure wave generated by breaching charges, high-calibre weapons, and explosives during training and operations.^3,4^ While single high-intensity blasts can cause moderate-to-severe traumatic brain injury (TBI), most exposures in active-duty SOF are lower in intensity, occur repeatedly, and fall below diagnostic thresholds for concussion or mild TBI.^5^ Growing evidence indicates that such cumulative, subconcussive exposures produce subtle yet progressive impairments in cognition, mood and performance, with potential long-term implications for neurodegenerative risk.^6,7^ Despite these emerging links, the underlying biological mechanisms remain insufficiently defined, and validated biomarkers for early detection or risk stratification are still lacking.^8^

In SOF breachers and snipers, cumulative BOP exposures can number in the hundreds to thousands over a career. Common symptoms—headache, tinnitus, slowed cognition, irritability, mood disturbance, memory changes and sleep disruption—are characteristic of chronic, low-grade neurological dysfunction.^2,9-12^ These complaints frequently coincide with elevated circulating markers of axonal and glial injury, indicating sustained neuroinflammation and early neurodegenerative change that often remains undetectable on routine neuroimaging^13-16^ Together, these observations highlight the need for multimodal tools that can detect subclinical brain injury in chronically BOP-exposed personnel, as the persistent symptom-biomarker profile points to an underlying degenerative process requiring sensitive *in vivo* diagnostic approaches for monitoring and informing early preventive or therapeutic interventions.^17^

Mechanistically, blast shockwaves impart complex shear, tensile and rotational forces on the brain, causing diffuse axonal injury, blood-brain barrier (BBB) disruption, oxidative stress and neuroinflammation.^16,18,19^ These baromechanical insults disrupt kinase-phosphatase homeostasis, driving aberrant tau phosphorylation (p-tau) and cytoskeletal destabilization.^20,21^ Hyperphosphorylated tau exhibits reduced microtubule affinity, detaches, misfolds and oligomerizes into aggregate tau filaments that assemble into neurofibrillary tangles (NFTs)—toxic aggregates that impair axonal transport, disrupt synaptic function and propagate trans-synaptically to promote cognitive decline.^22,23^ Blast-induced mitochondrial dysfunction and oxidative stress further amplify these effects, compromising glymphatic and proteostatic clearance.^24^ Concurrent BBB dysfunction allows immune infiltration, sustaining chronic glial activation and neuroinflammation.^16,25^ Together, these mechanisms may drive the co-occurrence of tau and amyloid-β abnormalities consistent with neuropathological reports in cases of blast-related neurotrauma.^21,26,27^

Neuropathological studies in animal models and blast-exposed Veterans describe perivascular accumulations of p-tau in neurons, astrocytes and cell processes at the depths of the cortical sulci, most prominently within superficial frontal and temporal cortices, with additional hippocampal and subcortical involvement.^28-30^ This lesion pattern, defined by NINDS/NIBIB consensus as pathognomonic, distinguishes chronic traumatic encephalopathy (CTE) from Alzheimer’s disease and other tauopathies.^31^ Although these lesions can resemble early CTE neuropathologic changes associated with repetitive head impacts, they may exhibit a distinct regional distribution, reflecting differential vulnerability to mechanical strain, neuroinflammation and vascular stress.^26,32,33^ Clinically, CTE manifests as progressive irritability, aggression, depression and memory loss that can evolve to dementia.^27,29^ While repetitive concussive trauma is clearly associated with tauopathy in contact sports, mechanistic links between cumulative low-level BOP and CTE-like neuropathology remain unclear.^34,35^ Direct *in vivo* evidence connecting cumulative occupational blast exposure to regionally specific tau deposition remains limited, particularly in well-characterized SOF cohorts.

Although CTE remains a neuropathological diagnosis confirmed post-mortem,^29^ advances in molecular imaging and fluid biomarker assays now permit earlier *in vivo* detection of tau pathology.^36,37^ Positron emission tomography (PET) using tau-selective radioligands such as [^18^F]flortaucipir (also known as [^18^F]T807/AV-1451) enables regional visualization and quantification of aggregated tau/NFTs, providing a sensitive means to assess blast-related neurodegenerative changes in living subjects.^38,39^ In blast-exposed individuals, tau-PET studies demonstrate elevated frontal and temporal uptake correlating with cognitive and neuropsychiatric symptoms, with up to half of repeatedly exposed Veterans showing abnormal cortical binding consistent with blast-related tauopathy.^20,40-42^ However, interpretation remains constrained by small sample sizes, heterogeneous exposures and limited active-duty SOF representation.

Parallel advances in plasma immunoassays now allow quantification of neuronal and astroglial injury markers—including phosphorylated tau isoforms, brain-derived tau (BD-tau), glial fibrillary acidic protein (GFAP), neurofilament light chain (NfL), ubiquitin C-terminal hydrolase-L1 (UCH-L1) and Aβ peptides.^43^ These minimally invasive biomarkers provide sensitive indices of tauopathy, axonal degeneration and amyloid dysregulation. Integrating tau-PET with plasma biomarker profiling offers a powerful multimodal approach for detecting blast-related neurodegeneration and supporting early risk stratification.^17,37^ Such combined strategies may elucidate the neurobiological sequelae of repetitive BOP exposure and identify individuals at heightened risk of progressive neurological dysfunction. Although prior military studies have reported blast-related alterations in brain structure and function, multimodal investigations combining tau-PET with fluid biomarkers in elite SOF cohorts are limited.^1,2,5,44^

In this cross-sectional study, we examined active-duty SOF personnel with extensive occupational BOP exposure compared with minimally exposed military controls. We integrated [^18^F]flortaucipir PET, structural MRI, ultrasensitive plasma biomarker profiling and standardized clinical assessments to test whether cumulative BOP exposure is associated with (i) increased regional cortical tau deposition, particularly within frontal and temporal cortices and (ii) plasma biomarker signatures indicative of early tauopathy, astroglial activation and axonal injury. We further hypothesized that regional tau burden would correlate with circulating markers of neuronal and glial injury, as well as with self-reported cognitive and neuropsychological symptoms. By linking molecular, neuroimaging, and behavioural domains, this study aimed to define early correlates of blast-related tau pathology to inform future longitudinal investigations of neurodegenerative risk in chronically exposed personnel.

## Materials and methods

### Study design and oversight

This cross-sectional observational study was conducted between March 2020 and June 2023 at the Brain Health Imaging Centre, Centre for Addiction and Mental Health (CAMH), Toronto, Canada. The protocol was approved by the CAMH Research Ethics Board and the Defence Research and Development Canada (DRDC) Human Research Ethics Committee. All procedures complied with the Declaration of Helsinki and the Canadian Tri-Council Policy Statement on Ethical Conduct for Research Involving Humans. Written informed consent was obtained from all participants before enrolment. The study was designed to examine the association between cumulative occupational BOP exposure in active-duty SOF personnel and *in vivo* evidence of tauopathy, complemented by plasma biomarker analyses of neuronal and astroglial injury.

### Participants

Two groups of male Canadian Armed Forces (CAF) personnel were recruited. The blast-exposed cohort included 25 active-duty SOF members with extensive BOP exposure, defined as ≥16 years of service in roles involving explosive breaching, munitions handling and/or frequent firing of high-calibre or shoulder-fired weapons. The control group comprised 10 age-matched CAF personnel from non-SOF units with minimal lifetime blast exposure, operationally defined as <3 cumulative years in occupations involving breaching or explosives-related activities.

All participants underwent [^18^F]flortaucipir PET, structural MRI, computerized neurocognitive testing and venous blood collection for plasma biomarker analysis. Demographic and military service data were documented, including total years of service, cumulative months deployed in combat zones and years in breaching or explosives-related roles. Lifetime blast exposure histories were obtained using structured interviews administered by trained study personnel.

### Eligibility and screening

Eligible participants were required to be active-duty military personnel aged 19 years or older. Exclusion criteria included a history of moderate or severe TBI; major neurological disorders such as epilepsy or multiple sclerosis; current substance use disorder; or major psychiatric illness other than stable post-traumatic stress disorder (PTSD), attention-deficit/hyperactivity disorder, depression or anxiety. Contraindications to MRI or PET (e.g. metallic implants, severe claustrophobia) were also exclusionary.

## Clinical and neurobehavioural assessments

### Participant history

A structured, in-person clinical intake was completed by trained research personnel to document each participant’s medical, neurological and psychiatric history; current medications; and recreational drug and alcohol use. The assessment also captured prior head injuries, episodes of loss of consciousness, deployment history and persistent post-concussive symptoms. Military service information, including years of service, months deployed and occupational speciality, was obtained through structured self-report. Blast exposure history was quantified using a standardized interview protocol, capturing years spent in breaching, sniping, or explosive ordnance roles, estimated number of blast events, and cumulative months deployed. These variables were integrated to derive a proxy index of cumulative BOP exposure.

### Symptom, function and cognitive measures

Participants completed validated self-report instruments previously used in CAF research (Table S1) to assess symptom burden across domains relevant to blast-related neurotrauma.^9,10^ These included the Rivermead Post-Concussion Questionnaire (RPQ), PTSD Checklist for DSM-5 (PCL-5), Pittsburgh Sleep Quality Index (PSQI), Beck Depression Inventory-II (BDI-II), Patient Health Questionnaire-9 (PHQ-9), Generalized Anxiety Disorder-7 (GAD-7), 36-Item Short Form Health Survey (SF-36) and the Military Concussion Readiness Inventory-Dismounted Battlefield (MCRI-DB).

A 45-minute computerized cognitive battery assessed domains sensitive to blast-related dysfunction,^10^ including short-term visual recognition memory (Delayed Matching to Sample), sustained attention and psychomotor speed (Four-Choice Reaction Time), working memory (N-Back: 1-, 2-, and 3-back), and executive control (Stroop Interference). Performance metrics—accuracy, mean response latency and load-related changes—were recorded automatically. Neurocognitive outcomes were subsequently compared with regional tau-PET uptake and plasma biomarker concentrations.

## Neuroimaging acquisition and processing

### Positron emission tomography

All PET scans were acquired on a GE Discovery MI PET/CT scanner (GE Healthcare, Waukesha, WI, USA) following established [^18^F]flortaucipir imaging protocols. The radioligand was synthesized on-site at CAMH and administered intravenously as a bolus of 185 MBq (±10%; 5 mCi). After an uptake period of approximately 45 min in a quiet, dimly lit room, PET acquisition commenced with a low-dose CT for attenuation correction, followed by 75 min of dynamic list-mode PET. A custom-fitted thermoplastic face mask minimized head motion. PET data were reconstructed using filtered back projection with ramp and Hanning filters at the Nyquist cutoff. Frames (6 × 300 s) yielded a final image matrix of 256 × 256 × 207 voxels (1.2 × 1.2 × 1.2 mm^3^; field of view & 312 × 312 × 253 mm^3^). Standard corrections for decay, randoms, dead time, normalization, attenuation and scatter were applied.

### Magnetic resonance imaging

High-resolution structural MRI was acquired on a 3 T GE Discovery MR750 scanner using a 32-channel head coil (Nova Medical Inc., Model 3832016, USA). A spoiled gradient-echo sequence was used with the following parameters: echo time (TE) = 17 ms, repetition time (TR) = 6000 ms, field of view = 220 mm, matrix = 256 × 256 × 200 voxels, voxel size = 0.9 × 0.9 × 2 mm^3^ and number of excitations = 2.

### Image processing and VOIs

PET and MRI data were processed using PMOD 4.2 (PMOD Technologies Ltd, Zurich, Switzerland) and SPM12 (Wellcome Centre for Human Neuroimaging, UCL). Dynamic PET frames were motion-corrected by rigid-body realignment and co-registered to each participant’s T1-weighted MRI. Volumes of interest (VOIs) were defined using the Hammers probabilistic atlas. T1-weighted images were segmented into grey matter, white matter and cerebrospinal fluid, then normalized to MNI space in SPM12. The inverse deformation field was applied to extract time-activity curves from each anatomical VOI. Mean radioactivity concentration (kBq/mL) was calculated over the late post-injection interval (45–75 min), corresponding to the equilibrium phase of [^18^F]flortaucipir binding.

Standardized uptake value ratios (SUVRs) were computed by dividing each target VOI SUV by that of the cerebellar cortex, chosen as a reference region for its relative resistance to tau deposition in non-AD tauopathies. Six cortical VOIs—frontal, temporal, parietal, occipital, insular and anterior cingulate cortices—were included in the primary analysis.

### Voxelwise analysis

To identify regions of elevated [^18^F]flortaucipir binding in blast-exposed participants, voxelwise SUVR parametric maps were generated by normalizing each voxel to the cerebellar reference. For each exposed participant, a voxelwise *Z*-score map was computed relative to the control group (*n* = 10): *Z_i_* = (*x_i_* − *μ*)/*σ*, thresholded at *Z* ≥ 1.96 (*P* < 0.05, two-tailed). MRI-derived brain masks removed extracerebral signal and regions of known non-specific binding. Clusters were extracted in MATLAB (R2022a) using *regionprops3* with an 18-connected neighbourhood. For each cluster, centroid coordinates, volume (mL), and mean/range SUVR and *Z* values were computed. Clusters <0.11 mL (≥62.5 voxels at 1.2 mm isotropic; ≈0.8 × 0.8 × 0.8 cm^3^) were excluded to maintain sensitivity across subjects. The minimum SUVR among retained clusters was recorded as a potential lower bound for elevated uptake in this cohort.

### Plasma biomarker analysis

Peripheral venous blood was collected into 10 mL K_2_EDTA tubes on the day of PET imaging. Samples were allowed to rest at room temperature for ∼45 min, then centrifuged at 1300 *g* for 20 min at 4°C. Plasma was aliquoted into polypropylene tubes and stored at −80°C until analysis.

Plasma concentrations of ten neurological biomarkers were quantified using the ultrasensitive single-molecule array (SiMoA^®^ HD-X; Quanterix, Billerica, MA, USA) platform. Tau-related neuroproteins included total tau (t-tau), brain-derived tau (BD-tau) and phosphorylated tau isoforms p-tau181, p-tau217 and p-tau231. Additional analytes comprised glial fibrillary acidic protein (GFAP), neurofilament light chain (NfL), ubiquitin C-terminal hydrolase-L1 (UCH-L1), and amyloid-β_1–42_ (Aβ42) and amyloid-β_1–40_ (Aβ40); the Aβ42/40 ratio indexed relative amyloid processing.

All assays were performed in duplicate within a single analytical batch using commercially available kits (Neurology 4-Plex, p-Tau Advantage and BD-Tau Advantage PLUS) according to manufacturer protocols. Sample, internal, inter-plate, and negative controls were included to verify assay performance and maintain quality control. Laboratory personnel were blinded to participant group assignments, and mean duplicate values were used in all analyses. Intra- and inter-assay coefficients of variation were <20% for all biomarkers, with all concentrations falling within manufacturer-specified detection limits.

### APOE4 genotyping

Genomic DNA was extracted from venous blood using a standard high-salt precipitation method. Genotyping for rs429358 and rs7412 (defining apolipoprotein E [*APOE*] isoforms) used TaqMan^®^ 5′ nuclease allelic discrimination assays (Applied Biosystems Inc.) on 96-well plates per manufacturer protocols. *APOEε4* carrier status was identified in only one participant (1/35); given the low prevalence, genotype data were not included in analyses.

### Statistical analysis

All analyses were conducted using IBM SPSS Statistics (Version 29.0; IBM Corp.), R (Version 4.3.2) and GraphPad Prism (Version 10.5.0). Continuous variables were assessed for normality using the Shapiro–Wilk test and are reported as mean ± SD or median (interquartile range, IQR), as appropriate; categorical variables are summarized as counts and percentages. Between-group differences were evaluated with independent-samples *t*-tests (parametric) or Mann–Whitney U tests (non-parametric) for continuous data, and χ^2^ or Fisher’s exact tests for categorical data. Effect sizes (Cohen’s *d*) and 95% confidence intervals (CIs) were computed for all continuous variables to quantify standardized between-group differences.

For comparisons including covariates (e.g. age), analysis of covariance (ANCOVA) was applied. Group differences in plasma biomarker concentrations were examined using Mann–Whitney U tests followed by Benjamini–Hochberg false discovery rate (FDR) correction within the biomarker family (*q* < 0.05 considered significant). All tests were two-tailed, with statistical significance defined as *P* < 0.05 unless otherwise adjusted.

Regional [^18^F]flortaucipir SUVRs across six cortical VOIs were analysed using repeated-measures ANCOVA, with group (SOF versus control) as the between-subject factor and VOI as the within-subject factor, adjusting for age. Sphericity was assessed with Mauchly’s test, and Greenhouse–Geisser correction applied where violated. Planned *post hoc* contrasts focused on frontal and temporal cortices with Bonferroni-adjusted *α* = 0.025.

Associations among cumulative blast exposure metrics (years in breaching, years handling explosives, estimated number of explosive events), plasma biomarkers, neurobehavioural measures, and regional tau-PET uptake were examined using multivariable linear regression models adjusting for age, education, total years of military service, and prior head-injury history. Model assumptions of linearity, normality and homoscedasticity of residuals were verified, and multicollinearity was assessed using variance inflation factors (VIF < 2 deemed acceptable). Associations were considered robust only if they remained statistically significant across these sensitivity analyses. Standardized *β* coefficients and 95% CIs were reported for all significant associations. For exploratory analyses, Pearson or Spearman correlations were computed according to data distribution.

## Results

### Participant characteristics

Descriptive characteristics of the study sample are presented in Table 1. Thirty-five male CAF personnel participated: 25 active-duty SOF members with extensive occupational blast exposure and 10 non-SOF controls with minimal lifetime exposure. Groups were range-matched for age within ± 5 years, yielding mean (SD) ages of 43.6 (6.1) years for the SOF group and 39.8 (6.8) years for controls. Although SOF participants were slightly older, this difference was not statistically significant after accounting for matching criteria.

**Table 1:** Participant characteristics by blast-exposure groups

Characteristic	SOF (*n* = 25)	Controls (*n* = 10)	*P* value	Cohen’s *d* (95% CI)
**Demographics**				
Age, year (mean ± SD)	43.6 ± 6.1	39.8 ± 6.8	0.092	0.59 (−0.09 to 1.26)
BMI, kg/m^2^	27.8 ± 3.9	25.4 ± 3.1	0.038	0.68 (0.01–1.36)
Years of education	13.6 ± 1.8	15.4 ± 2.1	0.019	−0.91 (−1.59 to −0.23)
Race, no. (%)				
Asian	1 (4)	0 (0)	–	–
Black	0 (0)	1 (10)	–	–
White	22 (88)	9 (90)	0.86^a^	–
Hispanic	1 (4)	0 (0)	–	–
Mixed/multiple	1 (4)	0 (0)	–	–
**Substance use**				
Current smokers no. (%)	3 (12)	1 (10)	0.87^a^	–
Cannabis use (past 30 days) no. (%)	6 (24)	0 (0)	0.04^a^	–
Alcohol use, drinks/week mean (range)	5.6 (3.2–9.8)	4.8 (2.4–6.2)	0.39	0.26 (−0.42 to 0.93)
**Psychiatric history**				
DSM-5 diagnosis no. (%)	9 (36)	2 (20)	0.42^a^	–
**Military service and exposure**				
Years of service	21.8 ± 4.9	12.4 ± 4.7	<0.001	1.97 (1.13–2.81)
Months in Combat deployment	27 (12–48)	8 (0–18)	0.002	1.23 (0.58–1.89)
Years in breaching	12.6 ± 5.7	0.4 ± 0.8	<0.001	2.75 (1.72–3.78)
Years with explosives	10.8 ± 4.6	0.3 ± 0.6	<0.001	2.91 (1.82–4.00)
Time since last blast (days)	114 (36–298)	–	<0.001	1.34 (0.65–2.03)
Time since last blast <1 year no. (%)	19 (76)	1 (10)	<0.001^a^	–
**Injury history no. (%)**				
Head injury	23 (92)	3 (30)	<0.001^a^	–
Head trauma with LOC	11 (44)	0 (0)	0.011^a^	–
Motor vehicle accident	14 (56)	3 (30)	0.17a	–
Physical fights	10 (40)	3 (30)	0.56a	–
Childhood falls	18 (72)	4 (40)	0.06a	–
**Behavioural questionnaires**				
BDI-II score	14.2 (8.6–21.5)	7.4 (5.8–9.6)	0.013	0.89 (0.23–1.55)
PHQ-9	9.8 ± 5.4	5.6 ± 3.1	0.018	0.90 (0.23–1.57)
GAD-7	8.9 ± 4.8	5.1 ± 2.9	0.022	0.85 (0.18–1.52)
MCRI-DB	48.7 ± 10.3	61.8 ± 7.2	<0.001	−1.44 (−2.16 to −0.72)
RPQ	18.2 (12.6–25.9)	7.5 (5.1–10.8)	0.009	1.05 (0.38–1.72)
PCL-5	32.5 (21.3–47.4)	18.7 (12.2–23.1)	0.021	0.91 (0.25–1.57)
PSQI	9.1 ± 3.8	5.2 ± 2.1	0.006	1.17 (0.48–1.86)
SF-36 (QOL)	62.4 ± 8.3	74.5 ± 7.6	<0.001	−1.55 (−2.29 to −0.81)
**Neurocognitive performance**				
4-Choice RT accuracy (%)	91.6 ± 6.3	96.8 ± 3.1	0.007	−1.09 (−1.76 to −0.42)
DMTS accuracy (%)	87.3 ± 7.2	93.4 ± 3.8	0.011	−0.98 (−1.64 to −0.31)
Stroop interference RT (ms)	645 (588–701)	588 (550–612)	0.028	0.77 (0.12–1.42)
N-back D′ (2-back)	1.72 ± 0.38	2.14 ± 0.41	0.009	−1.04 (−1.71 to −0.37)

Values are mean ± SD unless otherwise indicated. Cohen’s *d* (95% CI) quantifies standardized between-group differences.

BMI, body mass index; LOC, loss of consciousness; RT, reaction time; SOF, special operations forces; QOL, quality of life.

Superscripts denote statistical tests used:

^a^χ^2^ or Fisher’s exact test.

Operational histories differed markedly between groups. SOF personnel reported significantly longer total military service, greater cumulative months deployed in combat zones, and substantially more years in breaching or explosives-related roles (all *P* < 0.001). The interval since last blast exposure was significantly shorter in the SOF group. Compared with controls, SOF participants also exhibited higher body-mass index (BMI), fewer years of formal education and a higher prevalence of self-reported cannabis use.

A lifetime history of head injury was reported by 92% of SOF personnel versus 30% of controls (*P* < 0.001). Head injuries involving loss of consciousness occurred in 44% of SOF participants and in none of the controls (*P* = 0.011). SOF members additionally reported more frequent childhood falls. Clinically, they endorsed greater depressive symptoms, higher post-concussive symptom burden, poorer sleep quality and lower operational readiness scores.

Large between-group differences were evident across several operational and clinical variables. The strongest effects were observed for cumulative service exposure—years in breaching or explosives roles (*d* = 3.84 [95% CI, 2.74–4.94]) and total years of service (*d* = 1.64 [1.03–2.25])—as well as for operational readiness (*d* = −1.82 [−2.43 to −1.21]). Moderate-to-large effects were also present for post-concussive symptom burden (*d* = 1.69 [1.04–2.34]) and PTSD symptoms (*d* = 1.64 [0.98–2.30]). Smaller but directionally consistent effects were observed for education, BMI and sleep quality.

## Neuroimaging

### Group differences in regional tau-PET uptake

**Figure 1:**
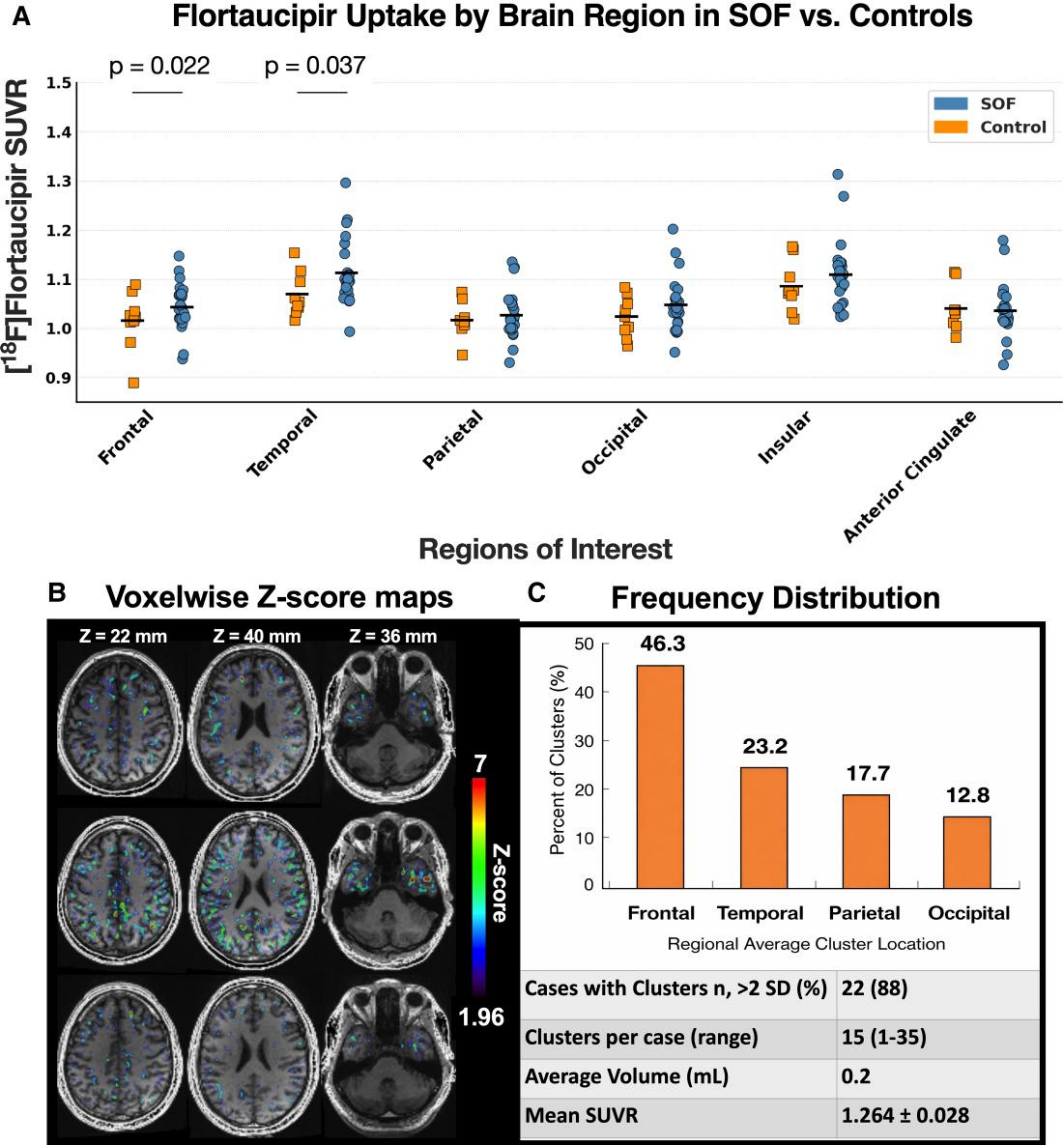
**Regional and voxelwise [^18^F]flortaucipir uptake in blast-exposed SOF and controls.** Cerebellum-referenced standardized uptake value ratios (SUVRs) across six cortical regions of interest (ROIs; **A**) frontal, temporal, parietal, occipital, insular, and anterior cingulate cortices—in blast-exposed SOF (*n* = 25; blue circles) and unexposed controls (*n* = 10; orange squares). Horizontal bars indicate group medians. A repeated-measures analysis of covariance (ANCOVA) adjusted for age showed a significant group × region interaction (*P* = 0.024). *Post hoc* Bonferroni-corrected tests demonstrated higher SUVRs in SOF within the frontal (*P* = 0.022) and temporal (*P* = 0.037) cortices. Voxelwise *Z*-score maps (**B**) overlaid on structural MRI horizontal slices from three representative SOF participants with moderate-to-high tau burden (top: 116 mL; middle: 48 mL; bottom: 36 mL cumulative suprathreshold cluster volume, **B**). Greater tau load corresponds to larger deviations from control values (mean SUVR across these cases = 1.21). *Z*-score maps (threshold > 1.96; >2 standard deviations [SD] above control mean) identify cortical locations with elevated [^18^F]flortaucipir binding. Colour scale denotes the number of SOF participants with suprathreshold uptake at each voxel. Clusters of ≥62.5 contiguous voxels were observed in 22 of 25 SOF participants. Frequency distribution of suprathreshold clusters by lobar region (**C**). Inset: proportion of SOF with clusters (88%), median cluster count per case = 15 (range = 1–35), mean cluster volume = 0.2 mL, and mean SUVR = 1.264 ± 0.028.

As shown in Fig. 1A, regional [^18^F]flortaucipir SUVRs were compared between SOF members with repetitive BOP exposure (*n* = 25) and controls (*n* = 10) across six cortical regions of interest (ROIs: frontal, temporal, parietal, occipital, insular and anterior cingulate). Repeated-measures ANCOVA revealed a significant main effect of group (*F*(1, 33) = 4.84, *P* = 0.035) and a significant ROI × group interaction (*F*(3.14, 103.65) = 3.21, *P* = 0.024). When age was included as a covariate, both effects were attenuated (*F*(1, 32) = 3.36, *P* = 0.046; *F*(3.42, 109.46) = 1.97, *P* = 0.114). Age-adjusted pairwise comparisons demonstrated significantly higher mean [^18^F]flortaucipir SUVRs in the frontal cortex of blast-exposed SOF participants relative to controls (mean difference = + 4.7%, *P* = 0.022), which remained significant after Bonferroni correction for planned comparisons. A similar pattern was observed in the temporal cortex (mean difference = + 5.3%, *P* = 0.037), although this effect did not survive correction for multiple comparisons. Exploratory analyses indicated non-significant trends towards elevated uptake in the occipital (*P* = 0.059) and parietal (*P* = 0.080) cortices, whereas insular (*P* = 0.283) and anterior cingulate (*P* = 0.782) regions showed no group differences.

### Voxelwise mapping of elevated tau signal

Voxelwise *Z*-score mapping revealed frontal-dominant [^18^F]flortaucipir uptake among blast-exposed SOF participants. Suprathreshold clusters, defined as regions exceeding 2 SDs above the control group mean and comprising ≥62.5 contiguous voxels (*Z* ≥ 1.96), were identified in 22 of 25 individuals (88%). Across these 22 participants, a total of 328 discrete clusters were detected. Nearly half (46.3%) were localized to the frontal lobes, followed by temporal (23.2%), parietal (17.7%), and occipital (12.8%) regions. The number of clusters per participant ranged from 1 to 35 (median & 15), with a mean cluster volume of 0.20 mL. One SOF participant exhibited several larger, confluent clusters. The mean SUVR within clusters was 1.24 ± 0.03 (range: 1.17–1.31). Many clusters were distributed along perivascular and sulcal boundaries, a pattern consistent with neuropathological reports of blast-associated tauopathy (Fig. 1B and C).

### Associations between cumulative blast exposure and tau deposition

**Figure 2:**
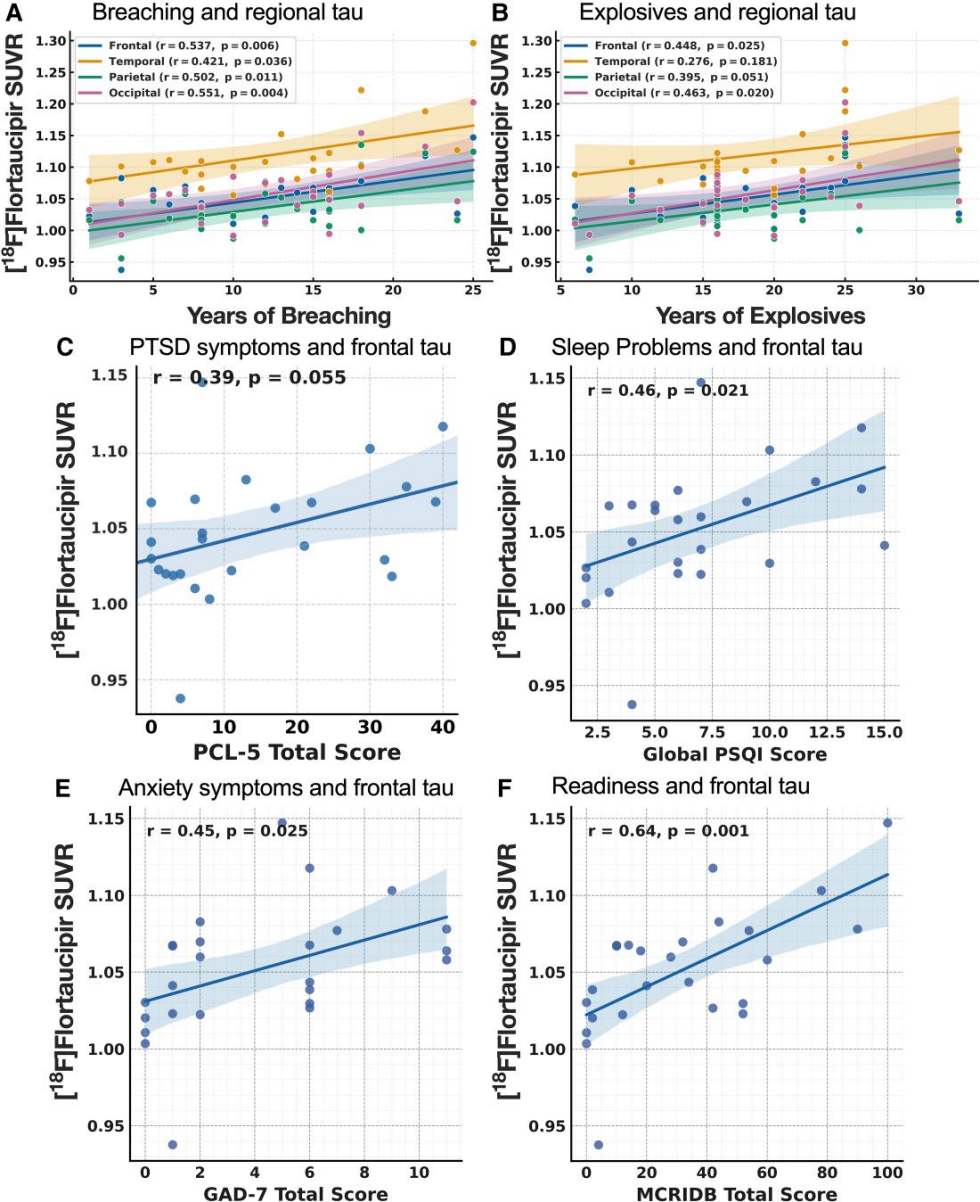
**Associations between cumulative blast exposure, neurobehavioural measures, and regional [^18^F]flortaucipir uptake in SOF.** Panels depict age-adjusted Pearson correlations between regional [^18^F]flortaucipir standardized uptake value ratios (SUVRs; cerebellar reference) and cumulative blast exposure metrics or clinical symptom scores in blast-exposed SOF participants (*n* = 25). Solid lines indicate fitted slopes; shaded bands represent 95% confidence intervals (CIs). (**A**, **B**) Greater years of breaching exposure and years of explosives use were associated with higher SUVRs, most prominently in the frontal cortex, with breaching exposure also significant in parietal and occipital regions. (**C**, **F**) Higher post-traumatic stress symptoms (PTSD Checklist for DSM-5 [PCL-5]; **C**), sleep disturbance (Pittsburgh Sleep Quality Index [PSQI]; **D**), and anxiety symptoms (Generalized Anxiety Disorder-7 [GAD-7]; **E**), and lower post-blast operational readiness (Military Concussion Readiness Inventory-Dismounted Battlefield [MCRI-DB]; **F**) each corresponded to greater frontal-cortex SUVR, indicating that higher symptom burden and reduced readiness were associated with higher regional tau deposition.

Cumulative occupational blast exposure was positively associated with regional [^18^F]flortaucipir uptake. In the combined sample, frontal SUVRs correlated with years of breaching exposure (*r* = 0.510, *P* = 0.009) and years of explosives use (*r* = 0.461, *P* = 0.018) (Fig. S1). In the SOF cohort, higher cumulative breaching exposure demonstrated significant positive associations with age-adjusted regional SUVRs (Fig. 2A) in the frontal (*r* & 0.537, *P* & 0.006), temporal (*r* & 0.421, *P* & 0.036), parietal (*r* & 0.502, *P* & 0.011), occipital (*r* & 0.551, *P* & 0.004) and cortices, whereas explosives exposure correlated with age-adjusted SUVRs in the frontal (*r* & 0.448, *P* & 0.025), parietal (*r* & 0.395, *P* & 0.051) and occipital (*r* & 0.463, *P* & 0.020) cortices.

### Symptom and functional correlates of tau deposition

Higher frontal [^18^F]flortaucipir uptake was associated with greater symptom burden across multiple domains. In the combined sample, frontal SUVRs correlated positively with PTSD severity (PCL-5; *r* & 0.47, *P* & 0.017), sleep disturbance (PSQI; *r* & 0.45, *P* & 0.021), and anxiety symptoms (GAD-7; *r* & 0.43, *P* & 0.027), and negatively with operational readiness (MCRI-DB; *r* & −0.49, *P* & 0.013) (Fig. 2C–F). Within the SOF group, higher SUVRs in the frontal, parietal and occipital cortices were significantly associated with greater post-concussive symptom burden (*P* = 0.009–0.015), poorer sleep quality (*P* = 0.009–0.010) and reduced duty readiness (*P* < 0.001–0.018). Associations with PTSD and anxiety were weaker and did not remain significant after correction (*P* = 0.021–0.056). Consistent with these findings, MCRI-DB scores also correlated strongly with cumulative breaching exposure (all *P* < 0.001), suggesting that greater operational blast exposure and higher regional tau burden are linked to reduced functional readiness in chronically exposed personnel.

### Plasma biomarker findings

**Figure 3:**
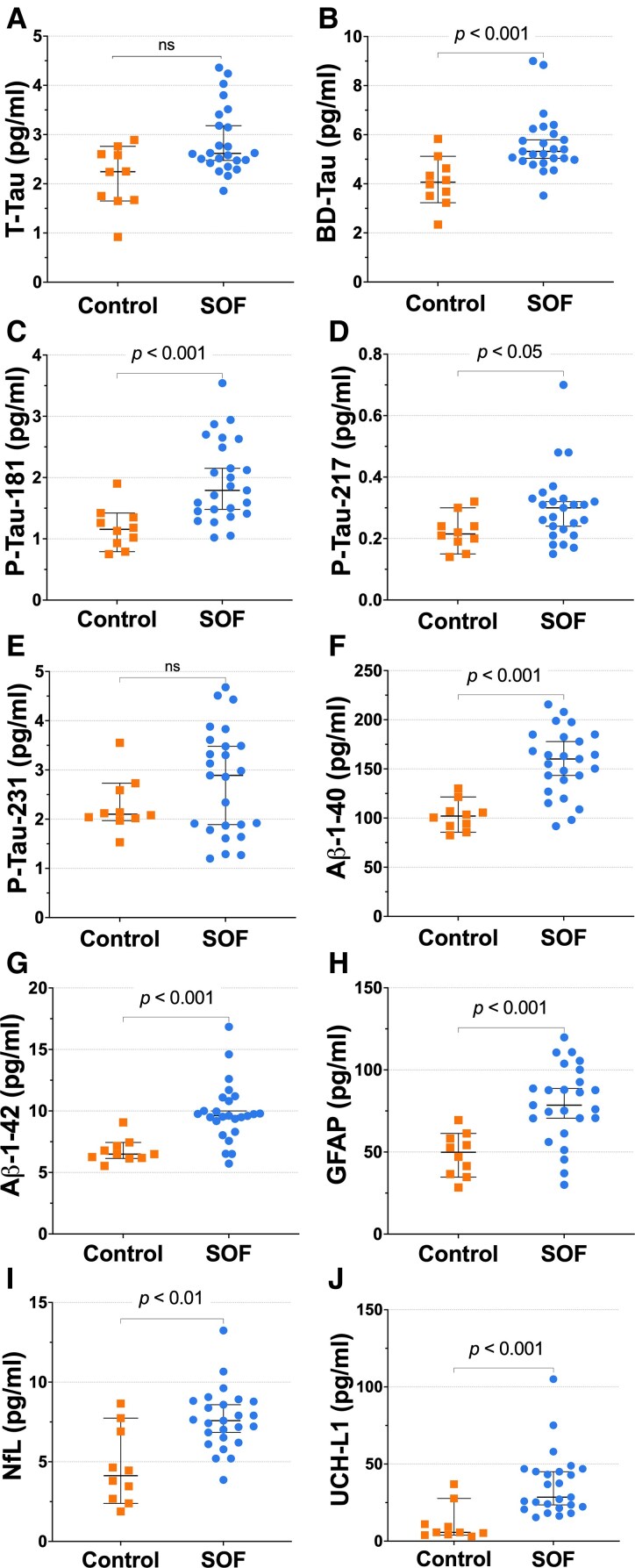
**Plasma biomarker concentrations in blast-exposed SOF and controls.** Group-wise plasma biomarker comparisons are shown for blast-exposed SOF (*n* = 25; blue circles) and minimally exposed military controls (*n* = 10; orange squares). Each point represents one participant; horizontal bars indicate group medians, and error bars show interquartile ranges (IQRs). Statistical significance was determined using Mann-Whitney U tests with FDR correction (*P* < 0.05); ‘ns’ = non-significant. (**A–C**) Markers of astroglial and axonal injury—GFAP, neurofilament light chain (NfL), and ubiquitin C-terminal hydrolase L1 (UCH-L1)—were significantly elevated in SOF participants. (**D–G**) Among tau-related biomarkers, p-tau181 and p-tau217 were higher in SOF, whereas t-tau and p-tau231 did not differ significantly. (**H**) Brain-derived tau (BD-tau) was markedly higher, reflecting increased CNS-specific tau release. (**I**, **J**) Amyloid-β peptides (Aβ_1–42_ and Aβ_1–40_) were also higher in SOF, accompanied by a reduced Aβ_42_/_40_ ratio (not shown). Together, these findings demonstrate that chronic, low-level blast exposure is associated with molecular evidence of astroglial activation, neuroaxonal injury, and disrupted tau- and amyloid-protein homeostasis in active-duty SOF personnel.

Plasma biomarker analyses revealed significant between-group differences across multiple indicators of neural injury, tauopathy and amyloid dysregulation (Fig. 3; Table S2). Compared with minimally exposed controls, SOF participants exhibited higher concentrations of GFAP (*P* & 0.001), NfL (*P* & 0.008) and UCH-L1 (*P* & 0.011), consistent with astroglial activation and axonal injury. Tau-related markers, including CNS-specific BD-tau (*P* & 0.002), p-tau181 (*P* & 0.012) and *p*-tau217 (*P* & 0.018), were likewise elevated, along with A-β_1–42_ (*P* & 0.006) and A-β_1–40_ (*P* & 0.009). The resulting reduction in the Aβ_42_/_40_ ratio (*P* & 0.040) suggests altered amyloid processing or impaired clearance. No significant group differences were observed for p-tau231 or t-tau, although both trended higher in SOF. All significant results remained robust following FDR correction across the biomarker family. Effect sizes (Cohen’s *d* & 0.9–1.3) indicated large, biologically meaningful group differences, supporting molecular evidence of astroglial activation, axonal injury and tau-amyloid dysregulation in chronically blast-exposed personnel.

### Associations between biomarkers, tau-PET uptake and clinical measures

In multivariate linear regression models adjusting for age, education, years of military service and head-injury history (*n* & 35), several plasma biomarkers were independently associated with regional [^18^F]]flortaucipir uptake (Table 2). GFAP showed the strongest associations with cortical SUVRs—frontal (*β* = 0.48, 95% CI 0.21–0.70, *P* = 0.006), temporal (*β* = 0.43, 95% CI 0.18–0.64), parietal (*β* = 0.41, 95% CI 0.15–0.62), and occipital (*β* = 0.46, 95% CI 0.19–0.67)—indicating that higher plasma GFAP was linked to greater regional tau deposition. BD-tau also demonstrated significant positive associations in the frontal (*β* = 0.37, 95% CI 0.09–0.61), temporal (*β* = 0.35, 95% CI 0.07–0.59), and parietal (*β* = 0.36, 95% CI 0.10–0.60) cortices (*P* = 0.015, FDR-corrected). Among amyloid-related markers, Aβ_42_ correlated with frontal (*β* = 0.44, 95% CI 0.18–0.65), parietal (*β* = 0.35, 95% CI 0.07–0.58), and occipital (*β* = 0.39, 95% CI 0.12–0.62) SUVRs (*P* = 0.012), while Aβ_40_ showed smaller but consistent effects (*β* ≈ 0.33–0.41, *P* = 0.028). p-tau181 was related to parietal (*β* = 0.33, 95% CI 0.06–0.56) and occipital (*β* = 0.35, 95% CI 0.10–0.59) uptake, whereas UCH-L1, a marker of axonal injury, showed positive associations with frontal, temporal and occipital regions (*β* ≈ 0.33–0.36, *P* = 0.037). No significant associations were observed for NfL, *p*-tau217 or p-tau231 after FDR correction.

**Table 2:** Associations between plasma biomarkers and regional [^18^F]flortaucipir SUVRs (*n* = 35)

Biomarker	Frontal *β* (95% CI)	Temporal *β* (95% CI)	Parietal *β* (95% CI)	Occipital *β* (95% CI)	FDR *P* adj.
GFAP	0.48 (0.21–0.70)	0.43 (0.18–0.64)	0.41 (0.15–0.62)	0.46 (0.19–0.67)	0.006^a^
BD-tau	0.37 (0.09–0.61)	0.35 (0.07–0.59)	0.36 (0.10–0.60)	0.40 (0.13–0.63)	0.015^a^
p-tau181	0.27 (–0.03–0.53)	0.19 (–0.08–0.46)	0.33 (0.06–0.56)	0.35 (0.10–0.59)	0.042^b^
p-tau217	0.02 (–0.27–0.28)	0.03 (–0.25–0.30)	0.09 (–0.18–0.34)	0.08 (–0.20–0.35)	ns
p-tau231	0.26 (–0.06–0.53)	0.15 (–0.11–0.43)	0.07 (–0.19–0.33)	0.04 (–0.22–0.30)	ns
Aβ40	0.39 (0.11–0.60)	0.41 (0.13–0.62)	0.28 (–0.02–0.52)	0.33 (0.04–0.56)	0.028^b^
Aβ42	0.44 (0.18–0.65)	0.36 (0.09–0.59)	0.35 (0.07–0.58)	0.39 (0.12–0.62)	0.012^a^
NfL	0.29 (0.01–0.54)	0.24 (–0.02–0.50)	0.18 (–0.09–0.44)	0.27 (–0.01–0.52)	ns
UCH-L1	0.36 (0.09–0.59)	0.34 (0.07–0.56)	0.28 (–0.01–0.52)	0.33 (0.04–0.57)	0.037^b^

Multivariate linear regressions adjusted for age, education, total years of military service, and head-injury history.

*β*, standardized regression coefficient; 95% CI, bias-corrected bootstrap confidence interval.

^a^ *P* < 0.01; ^b^*P* < 0.05 after FDR correction across biomarkers within each region.

Collectively, greater cortical tau burden in blast-exposed SOF personnel was most strongly linked to plasma indices of astroglial activation (GFAP), CNS-specific tau release (BD-tau) and amyloid dysregulation (Aβ_42_), with additional contributions from axonal injury (UCH-L1). These convergent molecular-imaging signatures were mirrored by clinical and functional associations (Table 3): higher regional [^18^F]flortaucipir uptake predicted greater post-concussive symptom burden (*β* = 0.48, *P* = 0.010), poorer sleep quality (*β* = 0.49, *P* = 0.012), increased PTSD and anxiety symptoms (*β* ≈ 0.38–0.42, *P* < 0.05) and lower operational readiness (*β* = −0.58, *P* = 0.004). Associations with depressive symptoms and general health were not significant after FDR correction. These findings suggest that repetitive low-level BOP exposure is associated with a coordinated pattern of glial and neuronal injury, cortical tau deposition and functional decline across multiple behavioural domains.

**Table 3:** Associations between regional [^18^F]flortaucipir SUVRs and clinical symptom measures in SOF (*n* = 25)

Clinical measure	Frontal *β* (95% CI)	Parietal *β* (95% CI)	Occipital *β* (95% CI)	Temporal *β* (95% CI)	FDR *P* adj.
GAD-7 (anxiety)	0.42 (0.08–0.65)	0.41 (0.07–0.63)	0.36 (−0.01–0.59)	ns	0.040^a^
PSQI (sleep quality)	0.49 (0.19–0.69)	0.47 (0.16–0.67)	ns	ns	0.012^b^
PCL-5 (PTSD)	0.38 (0.05–0.61)	0.42 (0.10–0.64)	0.37 (0.04–0.61)	ns	0.034^a^
RPQ (post-concussive)	0.48 (0.17–0.69)	0.45 (0.14–0.66)	0.44 (0.11–0.65)	ns	0.010^b^
MCRI-DB (readiness)	−0.58 (−0.75 to –0.30)	−0.44 (−0.68 to –0.13)	−0.49 (–0.71 to –0.22)	ns	0.004^b^
BDI-II/PHQ-9 (depression)	ns	ns	ns	ns	ns
SF-36 (general health)	ns	ns	ns	ns	ns

Models adjusted for age, education, total years of military service, and head-injury history.

*β*, standardized regression coefficient; 95% CI, bias-corrected bootstrap confidence interval; ns, non-significant.

^a^ *P* < 0.05; ^b^*P* < 0.01 after FDR correction across symptom scales within each cortical region.

## Discussion

Repeated exposure to BOP is increasingly recognized as a contributor to long-term neurodegenerative risk, yet the spatiotemporal trajectory of blast-related tau pathology in living service members remains insufficiently defined. In this first *in vivo* study of active-duty Canadian SOF personnel with extensive occupational blast histories, we found that chronic, low-level BOP exposure was associated with focal cortical tau accumulation and corresponding abnormalities in plasma markers of astroglial activation, axonal injury and amyloid dysregulation. Considered together, these convergent central molecular imaging and peripheral biomarker findings closely parallel post-mortem neuropathological changes documented in blast-exposed Veterans, suggesting that early biological features of blast-related neurodegeneration may be detectable during active military service.

Three principal findings emerged. First, SOF participants demonstrated elevated [^18^F]flortaucipir uptake in frontal cortex, and voxelwise analyses identified suprathreshold clusters in 88% of individuals, often located along perivascular and sulcal boundaries consistent with reported blast-associated tau pathology. Second, both regional tau-PET measures and circulating biomarkers, including BD-tau, GFAP and Aβ42, scaled with cumulative breaching and explosives exposure, supporting a dose–response relationship between occupational blast load and molecular indicators of neural injury. Third, greater frontal tau burden was associated with higher post-concussive and post-traumatic stress symptom severity, poorer sleep quality, mood disturbance and reduced operational readiness, underscoring the functional relevance of these biological alterations.

Collectively, these findings indicate that SOF personnel with prolonged, repeated low-level BOP exposure show a frontal-predominant pattern of tau deposition accompanied by glial, axonal and amyloid-related plasma abnormalities. Although these patterns parallel some post-mortem descriptions of blast-associated tauopathy, their detection in vivo underscores the need for longitudinal studies to determine their trajectory, clinical relevance and implications for long-term neurodegenerative risk in chronically exposed military populations.

### Blast exposure and regional tau deposition

The frontal- and temporal-predominant elevation of [^18^F]flortaucipir observed here aligns with neuropathological descriptions of blast-related tauopathy in Veterans^20,28,41,45,46^ and contrasts with the Braak progression of AD, in which tau first appears in transentorhinal cortex before spreading to hippocampal and neocortical regions.^38,47^ Comparative post-mortem brain studies confirm that regional [^18^F]flortaucipir retention reflects underlying tau deposition even at relatively low densities.^39,48^ Blast-associated tau typically exhibits a CTE-like distribution, with lesions in superficial cortical layers and perivascular zones of frontal and insular cortices,^30,49^ likely arising from biomechanical focusing of pressure waves along vascular channels and at sulcal depths.^21^ Given the prefrontal cortex’s central role in executive control, affect regulation and impulse modulation, such preferential involvement has clear operational and quality-of-life implications for SOF personnel.^6,7^

Although primary blast- and sport-related tauopathies share perivascular tau accumulations at sulcal depths, blast injury is driven by axial pressure-wave loading that produces selective perivascular stretch injury and frontal-predominant NFTs—distinct from the rotational-shear, diffuse sulcal involvement characteristic of blunt-impact and collision sports, and from the medial-temporal-to-neocortical progression typical of AD.^34,35^ These mechanistic and regional distinctions underscore the need for injury-specific biomarkers and precision imaging criteria in blast-exposed populations.^17,30^

Only a few prior studies have applied tau-PET in blast-exposed military populations. Dickstein *et al*.^20,50^ and Robinson *et al*.^42^ reported increased [^18^F]flortaucipir in frontal, parietal and temporal cortices of blast-exposed Veterans, while Chen *et al*.^40^ and Barrio *et al*.^51^ observed elevated [^18^F]FDDNP binding in frontal cortex and brainstem. Conversely, Gilmore *et al*.^5^ and Cummins *et al*.^52^ found no association between cumulative blast exposure and [^18^F]MK-6240 binding, likely reflecting the tracer’s preferential affinity for AD-type tau. De Bruin *et al*.^46^ demonstrated increased flortaucipir uptake after remote TBI, suggesting that trauma may accelerate otherwise age-related tau accumulation.

Our findings extend this literature in an operationally active SOF cohort with well-documented, high cumulative BOP exposure. We observed a robust frontal-dominant tau-PET pattern consistent with Veteran imaging.^20,41,42,50^ Voxelwise mapping revealed suprathreshold clusters in 88% of SOF participants, with nearly half in frontal cortex and many at sulcal depths or grey-white junctions, regions highly susceptible to blast-induced strain.^32,44^ The observed dose–response relationship between breaching/explosives exposure and frontal SUVRs further supports selective vulnerability of prefrontal circuits and reinforces the concept of a blast-related tauopathy distinct from AD.^27,30^

### Dose–response relationships with exposure intensity

A growing body of evidence supports a dose–response relationship between cumulative low-level BOP exposure and neurological dysfunction.^2,8,53^ Repeated exposures at or above ∼4 psi, particularly over years in high-impulse training contexts,^54,55^ have been linked to cognitive decline, persistent biomarker abnormalities and early tau pathology,^20,56,57^ with the largest effects reported in SOF personnel with extensive breaching histories.^9,13,58,59^ Our findings reinforce that chronic low-level blast and other subconcussive impacts can accelerate abnormal tau accumulation,^34,60,61^ supporting a mechanistic framework in which cumulative blast load contributes to progressive neurodegeneration.^21^

These findings also help contextualize the variability reported in post-mortem studies of blast-exposed individuals. Priemer^32^ identified CTE pathology in 4 of 45 blast-exposed brains, whereas Tripathy and colleagues^62^ observed no increased prevalence in a smaller Veteran cohort. More recently, Priemer *et al*.^61^ described severe stage IV CTE in a 44-year-old Naval Special Warfare Crewman with prolonged exposure to high-speed maritime impacts, despite the absence of known genetic risk factors. Together, these observations highlight the complexity of exposure–pathology relationships and the inherent limitations of retrospective autopsy studies, particularly when detailed lifetime exposure histories are incomplete or heterogeneous.

In contrast, the present operationally active cohort had well-documented, high-frequency BOP exposure, enabling a more direct evaluation of exposure–response relationships. The observed associations between cumulative breaching and explosive exposure and increased frontal [^18^F]flortaucipir SUVRs provide *in vivo* evidence linking quantified occupational blast load to region-specific tau deposition. The absence of a tau-PET association reported by Gilmore *et al*.^5^ using [^18^F]MK-6240 may reflect differences in tracer binding characteristics, exposure range, cohort composition and the lack of a truly unexposed comparison group. Nevertheless, their demonstration of altered network connectivity and microglial activation points to convergent circuit-level disturbances, even in the absence of elevated tau-PET signal.

### Symptom correlations underscore functional relevance

Associations between regional [^18^F]flortaucipir uptake and multiple symptom domains—including post-concussive complaints, sleep disruption, PTSD severity and reduced duty readiness—underscore the functional relevance of blast-related tau pathology.^2^ Elevated frontal, parietal and occipital signals mapped onto deficits in affect regulation, arousal control and visuospatial processing, consistent with prior observations in blast-exposed cohorts.^9,10^ The strongest and most consistent relationships were with the MCRI-DB,^63^ a validated indicator of field readiness, whose scores tracked closely with cumulative breaching exposure and regional tau burden.^5,27^

At the same time, limited associations with conventional neurocognitive tests (e.g. Stroop performance) mirror recent CAF data showing that snipers—not breachers—exhibited modest latent neurocognitive deficits.^64^ Together with Bigler’s critique that traditional neuropsychological instruments lack sensitivity to subtle and mechanism-specific mTBI effects,^65^ these findings caution against interpreting weak test correlations as evidence of preserved brain health. Rather, they highlight the value of multimodal assessment frameworks in which advanced imaging and biomarker measures detect neurobiological disruption that standard cognitive screens may miss.^59^ Overall, the pattern supports a model in which subclinical tau deposition contributes to a multidomain impairment profile resembling ‘Operator Syndrome’, encompassing co-occurring mood, sleep, neuroendocrine and cognitive disturbances.^1,66^

### Plasma biomarkers indicate convergent pathology

The plasma biomarker profile revealed convergent abnormalities across multiple pathways implicated in blast-related neurodegeneration. Elevated concentrations of BD-tau and phosphorylated tau species (p-tau181 and p-tau217) indicate CNS-specific tau release and dysregulated phosphorylation—processes that destabilize microtubules, impair axonal transport and facilitate aggregation into neurotoxic oligomeric and fibrillar species.^20,21^ The strong association between BD-tau levels and frontal tau-PET uptake supports the premise that circulating CNS-derived tau reflects underlying regional tau deposition.^67,68^ Notably, p-tau181 was elevated whereas p-tau231 remained unchanged—a pattern consistent with early-stage, non-Alzheimer tauopathy and aligned with peripheral p-tau profiles reported in blast-exposed cohorts.^5,69^

Astroglial activation was prominent, as evidenced by markedly elevated GFAP levels that showed the strongest and most consistent associations with cortical [^18^F]flortaucipir uptake across frontal, parietal and occipital regions.^70^ Under physiological conditions, astrocytes maintain ionic homeostasis, trophic signalling, synaptic integrity and cognitive function; following traumatic exposure, however, they adopt a reactive phenotype characterized by cellular hypertrophy, cytoskeletal remodelling and upregulation of GFAP.^71^ Circulating GFAP likely reflects astroglial injury in the setting of BBB disruption and/or impaired glymphatic clearance.^70^ Experimental and human studies demonstrate that BOP elicits GFAP-defined astrogliosis accompanied by neuroinflammation and neuronal injury, frequently co-occurring with microglial activation, vascular damage and metabolic stress.^19,72^ These responses preferentially localize to perivascular regions, grey-white matter junctions, and subpial layers—anatomical sites implicated in blast-related tau pathology.^33,35,61^ Consistent with this framework, low-level blast exposure in military cohorts is consistently associated with elevated circulating GFAP and, in some cases, the emergence of anti-GFAP autoantibodies.^2,15,56^ These observations, together with recent SOF PET-biomarker findings,^44^ support a model in which gliovascular injury and sustained reactive astrogliosis contribute to regionally selective tau aggregation following chronic blast exposure.^21,73^

Markers of axonal and synaptic injury (NfL, UCH-L1) were also elevated, in line with prior breacher/sniper studies.^56,74-76^ Although correlations with tau-PET were weaker than for GFAP or BD-tau, these elevations reflect ongoing axonal degeneration that destabilizes microtubules, impairs transport and promotes axonal-to-somatodendritic tau mislocalization—an early step in tangle formation.^23^ Persistent UCH-L1 elevations during breaching training^77^ and neuronal-derived extracellular vesicle tau correlating with symptom severity^57^ further support clinical relevance.

Finally, elevations in Aβ42 and Aβ40, together with a reduced Aβ42/40 ratio, point to dysregulated APP processing and/or impaired clearance.^56,78^ Blast exposure may impair perivascular and glymphatic pathways—via BBB transporter dysfunction (e.g. LRP1) or altered neuronal and astroglial mechanisms—promoting amyloid retention^79,80^ and potentially amplifying tau aggregation and neurotoxicity.^81^ Early amyloid PET signal in blast-exposed instructors^60^ and kinetic evidence linking cumulative BOP to plasma Aβ42 elevations^53^ support this interpretation. In Veterans, blast-related mild TBI has been associated with reduced CSF Aβ42/40 and a more frontal-parietal pattern of cortical amyloid deposition compared with typical AD.^45,46^

Apparent discrepancies between central and peripheral amyloid findings likely reflect the dual effects of blast exposure: acute neuronal release and peripheral spillover of Aβ species species,^16,59^ coupled with impaired perivascular and glymphatic clearance that alters CNS retention and spatial distribution.^82,83^ This profile contrasts with AD, in which plaque sequestration is typically associated with reduced circulating Aβ levels.^53^ Accordingly, Aβ measures, including the Aβ42/Aβ40 ratio, should be interpreted in conjunction with tau and neuroinflammatory biomarkers and contextualized by apolipoprotein E genotype and age. Impaired clearance and excess release may converge to promote amyloid–tau co-accumulation, exacerbate BBB dysfunction and reinforce a feed-forward cascade of neurodegeneration and cortical atrophy.^35^ Cumulative exposure appears to be a critical determinant: while a single mild blast can induce persistent neuroinflammation without overt structural injury,^28,50^ repeated blast exposures, particularly at higher impulse or peak pressures ≥4 psi, are associated with elevated p-tau, GFAP, amyloid alterations and increased frontal tau-PET signal in military cohorts.^2,5,20,57,59,76^ Our observed exposure–response relationships are consistent with these findings and support BOP as a distinct, environmentally driven trigger of tauopathy that may be amenable to targeted mitigation strategies.^8,49,84^

### Clinical and operational implications

The observed associations between frontal tau deposition and poorer sleep quality, heightened anxiety, greater PTSD symptom severity and reduced operational readiness underscore the functional relevance of blast-related neuropathology in active-duty personnel.^1,2,5^ Although causality cannot be inferred from this cross-sectional design, the convergence of neuroimaging, molecular and behavioural findings suggests that blast-related tau pathology may contribute not only to long-term neurodegenerative risk but also to near-term decrements in cognitive efficiency, affective regulation and operational performance.^7,85^ These observations are consistent with broader military literature demonstrating that subtle cognitive or psychological impairments may emerge even when conventional neuropsychological testing remains within normal limits,^65^ highlighting the added value of advanced neuroimaging and biomarker-based assessment frameworks.^9,10,17^

From an applied perspective, these findings support the development of a translational, exposure-informed surveillance model for personnel in high-risk occupations.^7,86^ The integration of tau-PET with ultrasensitive plasma biomarkers, including GFAP, BD-tau, and Aβ indices, offers a scalable approach to detecting subclinical neurobiological change, identifying individuals with high cumulative blast loads, and evaluating the effectiveness of blast-mitigation strategies.^36,59^ The increasing availability of field-deployable biomarker platforms further enables sampling in austere, training, or deployed environments, extending brain-health monitoring beyond traditional clinical settings.^87^

Operationally, such tools could support a tiered risk-management framework comprising baseline biomarker and neurobehavioural profiling during low-exposure periods, targeted reassessment following high-impulse training blocks or deployments, and intensified follow-up for individuals demonstrating biomarker elevations or emerging neurocognitive changes. Integration with cumulative BOP metrics would facilitate individualized return-to-duty decisions, inform career-long exposure thresholds and strengthen evidence-based policies aimed at preserving force readiness.^53,88^ Importantly, the strong associations observed between tau burden, sleep disruption and affective symptoms point towards potentially modifiable intervention targets. Optimizing sleep, mitigating stress and addressing mood disturbance may not only improve day-to-day functioning but could also plausibly influence downstream neurobiological trajectories. Collectively, these findings emphasize the need for proactive, data-driven approaches to brain-health monitoring in specialized military populations for whom repeated blast exposure is intrinsic to mission demands.^1,6^

### Limitations and future directions

Several limitations should be acknowledged. The modest sample size, constrained by operational demands and the availability of active-duty SOF personnel, reduced statistical power and precluded detailed subgroup analyses. The study cohort consisted almost entirely of white male participants, reflecting the demographic composition of the current Canadian SOF community. This demographic homogeneity limits the generalizability of the findings to broader and more diverse military and civilian populations, particularly given known sex-, gender- and race-related differences in tau biology, neuroinflammatory processes and neurodegenerative susceptibility.

The cross-sectional design further limits causal inference regarding the temporal sequence of blast exposure, tau deposition, biomarker perturbation and clinical outcome. While the observed dose–response associations are consistent with a causal mechanism, longitudinal investigations are required to determine whether the frontal-predominant tau pattern represents an early, potentially reversible stage or a biomarker of progressive neurodegeneration. Cumulative BOP exposure was estimated retrospectively using structured interviews corroborated by service records, rather than direct dosimetry, which limits exposure granularity. Future studies should incorporate wearable BOP dosimeters to provide precise, real-time exposure quantification and enable the development of refined exposure-response models.

Interpretation of [^18^F]flortaucipir PET findings must also consider potential off-target binding, including monoamine oxidase-B in the choroid plexus and basal ganglia. Our analyses therefore focused on cortical regions with minimal non-specific uptake. Second-generation tau tracers with greater sensitivity and specificity for non-AD and CTE-like tau conformations are expected to further enhance diagnostic precision and biological interpretability.

Moving forward, future research should expand molecular profiling to include novel markers of microglial activation, tau fragments (e.g. microtubule-binding region tau species with high aggregation potential) and synaptic dysfunction, and should integrate multimodal imaging approaches to capture convergent evidence of neurobiological injury. Longitudinal, multi-domain studies will be essential to validate early biomarkers, refine blast exposure models and guide preventive or therapeutic interventions aimed at preserving brain health in chronically exposed military populations.

## Conclusions

This study provides the first *in vivo* evidence that career-long, low-level BOP exposure in active-duty Canadian SOF personnel is associated with measurable frontal tau accumulation on [^18^F]flortaucipir PET, accompanied by convergent plasma biomarker abnormalities indicative of astroglial activation, CNS-specific tau release, axonal injury and amyloid dysregulation. The close correspondence between regional PET signal, circulating molecular markers and symptom domains, including sleep disturbance, post-concussive complaints and reduced operational readiness, highlights a clinically meaningful pattern of neurobiological alteration in chronically exposed operators. Beyond defining these biological correlates, our findings demonstrate the feasibility and translational value of integrating molecular neuroimaging with ultrasensitive blood-based biomarkers to detect subclinical brain injury during active service. Such multimodal approaches have direct implications for SOF brain-health surveillance, supporting individualized risk stratification, exposure-informed return-to-duty decisions and evaluation of blast-mitigation strategies. Longitudinal follow-up incorporating next-generation PET tracers and expanded biomarker panels will be essential to delineate temporal trajectories, establish clinical relevance and inform proactive, evidence-based brain-health policies for high-risk military occupations.

## Supplementary Material

fcag070_Supplementary_Data

## Data Availability

The datasets generated and/or analysed in this study involve active-duty Canadian Special Operations Forces personnel and are subject to strict privacy, security, and operational regulations. Consequently, raw data cannot be publicly shared. De-identified derived data may be made available to qualified investigators upon reasonable request to the corresponding author, contingent on institutional and governmental approvals and completion of a formal data use agreement.

## Supplementary material

Supplementary material (Tables S1 and S2) will be available at *Brain Communications* online.
