# Supplementary material for: Tau-PET imaging and blood biomarkers reveal early tauopathy in special operations forces exposed to repetitive blast
Source: Brain Commun. 2026 Mar 13;8(2):fcag070. doi: 10.1093/braincomms/fcag070 (PMC12993815; doi:10.1093/braincomms/fcag070)
Supplement: fcag070_Supplementary_Data [file fcag070_supplementary_data.docx]

**SUPPLEMENTARY TABLES**

**Table S1.** Summary of Questionnaires Administered

| Domain | Instrument Name | Description |
| --- | --- | --- |
| Mood and Depression | Patient Health Questionnaire (PHQ-9) | Assesses depressive symptom severity over the past 2 weeks. |
|  | Beck Depression Inventory (BDI) | Measures cognitive, affective, and somatic symptoms of depression. |
| Anxiety | Generalized Anxiety Disorder 7-item (GAD-7) | Screens for generalized anxiety symptoms. |
| Post-Traumatic Stress | PTSD Checklist (PCL-5) | Assesses PTSD symptoms based on DSM-5 criteria. |
| Sleep Quality | Pittsburgh Sleep Quality Index (PSQI) | Evaluates sleep quality and disturbances over the previous month. |
| Post-Concussive Symptoms | Rivermead Post-Concussion Symptoms Questionnaire (RPQ) | Captures common cognitive, emotional, and somatic symptoms following head trauma. |
| Functional Health and QoL | Short Form-36 Health Survey (SF-36) | Measures general health-related quality of life across physical and mental domains. |
| Vestibular/Balance Impairment | Military Concussion Readiness Inventory for Dizziness and Balance (MCRIDB) | Evaluates balance and dizziness symptoms in military personnel following head injury. |

**Table S2**. Plasma biomarker concentrations in blast-exposed Special Operations Forces (SOF) and controls

| **Biomarker (pg/mL)** | **SOF (n = 25)** | **Controls (n = 10)** | ***p* valueᵇ** | **Cohen’s d (95% CI)** |
| --- | --- | --- | --- | --- |
| GFAP | 122.4 (88.1–163.2) | 62.5 (50.4–79.9) | **<0.001** | 1.28 (0.67–1.89) |
| NfL | 29.7 (22.5–34.8) | 18.3 (15.7–22.0) | **0.008** | 1.12 (0.49–1.75) |
| UCH-L1 | 37.4 (29.8–45.1) | 24.9 (20.6–30.8) | **0.011** | 1.03 (0.41–1.65) |
| BD-tau | 3.8 (2.9–4.6) | 2.1 (1.8–2.5) | **0.002** | 1.24 (0.61–1.87) |
| t-tau | 2.9 (2.3–3.5) | 2.4 (1.9–2.9) | 0.089 | 0.53 (−0.05–1.11) |
| p-tau181 | 1.24 (1.01–1.54) | 0.84 (0.68–1.02) | **0.012** | 0.97 (0.36–1.58) |
| p-tau217 | 0.73 (0.59–0.92) | 0.51 (0.42–0.63) | **0.018** | 0.91 (0.31–1.51) |
| p-tau231 | 0.48 (0.40–0.59) | 0.43 (0.37–0.51) | 0.084 | 0.55 (−0.02–1.12) |
| Aβ₁-₄₂ | 27.8 (24.3–33.9) | 18.6 (16.8–22.4) | **0.006** | 1.15 (0.54–1.76) |
| Aβ₁-₄₀ | 238.9 (204.6–289.3) | 177.5 (158.7–199.6) | **0.009** | 1.10 (0.49–1.71) |
| Aβ₄₂/₄₀ ratio | 0.117 (0.109–0.122) | 0.123 (0.119–0.128) | **0.040** | −0.69 (−1.29––0.09) |

Values are median (IQR) in pg/mL. *p* values from Mann–Whitney *U* tests; Cohen’s d (95 % CI) quantifies standardized group differences. Bold values denote significance after FDR correction (q < 0.05). GFAP = glial fibrillary acidic protein; NfL = neurofilament light chain; UCH-L1 = ubiquitin C-terminal hydrolase-L1; BD-tau = brain-derived tau; t-tau = total tau; Aβ = amyloid-β.

**A**

**B**

**Figure S1.** Pearson correlations showing positive associations between mean [^18^F]flortaucipir Standard Uptake Value ratio (SUVr) in fontal, temporal, parietal and occipital cortices and cumulative blast exposure (A) years of breaching and (B) years of exposure to explosives in Special Operations Forces (SOF) and control sample (n = 35). Frontal SUVRs correlated with years of breaching exposure (r = 0.51, p = 0.009) and years of explosives use (r = 0.46, p = 0.018).
